# Alterations in Functional Constituents and Bioactivities of Tartary Buckwheat via Solid-State Fermentation with Three Edible-Medicinal Fungi: A Metabolomics-Based Analysis

**DOI:** 10.3390/foods14244187

**Published:** 2025-12-05

**Authors:** Qi Li, Jiaying Zhu, Xiangying Lv, Jin Liu, Hong Liu, Qingyuan Wang, Yunlong Li

**Affiliations:** 1 Shanxi Institute for Functional Food, Shanxi Agricultural University, Taiyuan 030031, China; 18792561959@163.com (Q.L.); z20223599@stu.sxau.edu.cn (J.Z.); 20232523@stu.sxau.edu.cn (X.L.); lj15234257497@163.com (J.L.); liuhong3089@126.com (H.L.); 2 Datong Tongtai Xin Beiqi Edible Fungi Development Co., Ltd., Datong 037000, China; 18935254139@163.com

**Keywords:** Tartary buckwheat, edible-medicinal fungi, non-targeted metabolomics, flavonoids, functional enhancement

## Abstract

To functionally enhance Tartary buckwheat and elucidate the underlying mechanisms of change, solid-state fermentation (SSF) was conducted using three edible-medicinal fungi—*Auricularia auricula* (*A. auricula*), *Ganoderma lucidum* (*G. lucidum*), and *Hericium erinaceus* (*H. erinaceus*). The in vitro antioxidant (DPPH/ABTS) and α-glucosidase inhibitory activities were quantitatively evaluated. Notably, SSF with *H. erinaceus* specifically elevated α-glucosidase inhibitory activity by 50% under the tested conditions. Non-targeted metabolomics further profiled metabolite alterations to identify key up-regulated bioactive compounds. Epicatechin gallate (ECG) was significantly up-regulated in all three samples, and the fold change in quercetin 3′-O-sulfate in GFTB was significantly higher than that in the other two samples. Metabolic pathway analysis identified the biosynthesis of secondary metabolites and the metabolism of terpenoids and polyketides as the most prominently affected pathways. This study demonstrates that SSF with edible-medicinal fungi is an effective bioprocessing strategy to boost the bioactivity and value of Tartary buckwheat.

## 1. Introduction

Tartary buckwheat (*Fagopyrum tataricum* (L.) Gaertn.), a member of the Polygonaceae family, is notable for its tolerance to low temperatures and barren soils, with widespread cultivation across the globe. Its seeds are abundant in essential nutrients including starch, proteins, and amino acids, while also containing unique bioactive compounds not found in other cereal crops such as flavonoids, along with phenolics, polysaccharides, and inositol [1]. Among these, phenolic compounds primarily exist in both free and bound forms. Most bound phenolics are covalently linked to plant macromolecules, rendering them insoluble and making it difficult to be fully utilized [2]. Conventional food processing methods, such as thermal treatments, often degrade the functional components of Tartary buckwheat [3]. In contrast, fermentation—a mild food processing technique with a millennia-long history of human use—plays a pivotal role in enhancing nutritional value, improving flavor, and boosting functional activity [4].

Solid-state fermentation (SSF) refers to the process in which solid substrates are fermented by microorganisms under conditions where there is almost no free water present [5]. Under these conditions, the metabolic activities of microorganisms significantly enhance the various phytochemical components and bioactivities in SSF products [6,7]. *Auricuralia auricula* (*A. auricula*), *Ganoderma lucidum* (*G. lucidum*), and *Hericium erinaceus* (*H. erinaceus*) are three edible-medicinal fungi commonly utilized in the food industry, combining safety and functionality. Among them, *A. auricula* ranks among the four major cultivated edible-medicinal fungi globally, boasting abundant bioactive substances such as polysaccharides, proteins, and melanin which exhibit immunomodulatory, antioxidant, hypoglycemic, and anti-inflammatory properties [8,9]. *G. lucidum,* a higher edible-medicinal fungus with a history exceeding 2000 years, contains a diverse array of functional components including polysaccharides, triterpenoids, nucleosides, and sterols [10], which possess anti-tumor, anti-aging, and antioxidant effects [11]. *H. erinaceus*, a well-known edible-medicinal fungus, boasts a diverse array of bioactive constituents, including steroids, polyunsaturated fatty acids, and polysaccharides [12]. Three edible-medicinal fungi species were selected for the SSF on their distinct functional properties and enzymatic capabilities. It was hypothesized that these differing metabolic potentials would lead to divergent biotransformation pathways of the Tartary buckwheat substrate, particularly in the modification of its flavonoid profile, allowing for a comparative analysis of their fermentation efficacy.

Using edible-medicinal fungi as fermentation strains for the SSF of grains can effectively enhance their nutritional and functional properties. Research has been conducted on the SSF of grains using edible-medicinal fungi: for instance, Kang et al. [13] found that SSF of buckwheat with *Agaricus* significantly increased its total phenol content (TPC) and antioxidant activity. Ren et al. [14] investigated the alcoholic extract of SSF with *Monascus purpureus* of Tartary buckwheat on the physique, blood lipid levels, blood glucose, and antioxidant enzyme activities in Kunming mice, demonstrating promising anti-obesity, hypoglycemic, and antioxidant capacities, suggesting its value as a dietary intervention for metabolic disorders. Nevertheless, existing research on SSF of Tartary buckwheat has primarily focused on nutritional and functional constituents, functional activities, and macro physiological utility evaluation, with few in-depth studies on metabolic changes during fermentation. Notably, the metabolic processes of flavonoids—Tartary buckwheat’s key functional substances—during SSF remain unsystematically investigated.

In this study, three edible-medicinal fungi were used for SSF of Tartary buckwheat to investigate alterations in its functional composition. We compared the effects of these fungi on the functional activity of Tartary buckwheat and analyzed their metabolites, particularly the changes in flavonoids during fermentation. This work intends to provide a theoretical groundwork for advancing the development and application of Tartary buckwheat as a functional food source.

## 2. Materials and Methods

### 2.1. Materials

Tartary buckwheat was sourced from Shanxi Yanmen Qinggao Food Industry Co., Ltd. (Datong, China). *A. auricula* (CICC 50036) and *H. erinaceus* (CICC 50081) were obtained from the China Center of Industrial Culture Collection (CICC), while *G. lucidum* (LH 676) was a laboratory stock. All strains were maintained on potato dextrose agar (PDA) slants: *A. auricula* and *H. erinaceus* were cultured at 25 °C for 10 days, while *G. lucidum* was incubated at 27 °C for 10 days and then refrigerated at 4 °C.

### 2.2. Chemicals and Reagents

Standard reagents (rutin, gallic acid) were purchased from the National Institute for Food and Drug Control (Beijing, China). Internal standard (2-Chloro-l-Phenylalanine) was purchased from Aladdin Biochemical Technology Co., Ltd. (Shanghai, China). p-Nitrophenyl-β-d-Galactopyranoside (PNPG) and α-glucosidase (from *Saccharomyces cerevisiae*) were purchased from Yuanye Biotechnology Co., Ltd. (Shanghai, China). 2,2′-Azinobis (3-ethylbenzothiazoline-6-sulfonic acid) (ABTS), folin-phenol and 2,2-Diphenyl-1-picrylhydrazyl (DPPH) were purchased from Solarbio Science & Technology Co., Ltd. (Beijing, China). Acetonitrile and methanol (both Liquid Chromatography–Mass Spectrometry (LC-MS) grade) were purchased from Anpel Experimental Technology Co., Ltd. (Shanghai, China). Formic acid (LC-MS grade) was purchased from Tokyo Chemical Industry Co., Ltd. (Tokyo, Japan). Methanol, anhydrous ethanol, and other analytical reagents were purchased from Sinopharm Chemical Reagent Co., Ltd. (Shanghai, China).

### 2.3. SSF of Tartary Buckwheat

A 50 g sample of Tartary buckwheat was subjected to hydration in four volumes (200 mL) of pure water over a 24 h period. Following the soaking phase, the water was drained and the buckwheat was sterilized via autoclaving (121 °C, 1 h) and subsequently cooled. Under aseptic conditions in a laminar flow cabinet, the sterilized material was inoculated with 5 mL aliquots of actively growing fungal cultures from different strains. The conical flasks were shaken thoroughly post-inoculation to mix the contents. SSF was carried out in erlenmeyer flasks covered with a breathable sealing film. Following solid-state fermentation, the resulting products were processed into a fine powder for subsequent analysis. The fermented Tartary buckwheat was first thoroughly dried, then mechanically pulverized. The powdered material was finally passed through an 80-mesh sieve to achieve a uniform particle size. The sample inoculated with *A. auricula* was designated AFTB, while those treated with *H. erinaceus* and *G. lucidum* were labeled HFTB and GFTB, respectively. The incubation conditions for the solid-state fermentation process were specifically optimized for each fungal species: both *A. auricula* and *H. erinaceus* were cultivated at 25 °C, whereas *G. lucidum* required a temperature of 27 °C. All fermentations were carried out for a consistent period of ten days. The control group (non-fermented Tartary buckwheat, named as NFTB) underwent the exact same thermal treatment (autoclaving at 121 °C for 1 h) and incubation conditions as the fermented groups, with the sole exception that no fungal inoculum was added. Extraction involved mixing 0.2 g of each sample, which was blended with 50 mL of 70% methanol and placed in a 70 °C water bath for 3 h to yield the sample extract. Following extraction, the resulting solution was stored in a light-shielded environment.

### 2.4. Determination of TPC

TPC was determined using the method described by Yan et al. [15]. Sample extracts were prepared according to the methodology outlined in Section 2.3. Concisely, 0.5 mL of the extract was transferred using a pipette, after which 0.8 mL of 0.1 mol·L^−1^ Folin-Phenol reagent and 2.7 mL of distilled water were introduced. This mixture was then incubated for 40 min in a water bath maintained at 40 °C. Subsequently, 2.5 mL of a 7% Na_2_CO_3_ solution was incorporated. The resulting reaction was kept in darkness for 6 min. The absorbance was ultimately recorded at 760 nm with an Ultraviolet-Visible spectrophotometer (A380, Aoyi Instruments Co., Ltd., Shanghai, China).

Standard curve plotting: The absorbance of gallic acid solutions with mass concentrations of 3.17, 6.33, 12.66, 19.00, and 25.33 μg·mL^−1^ was measured, and the gallic acid standard curve was constructed, yielding the regression equation Y = 19.042x + 0.0025 (R^2^ = 0.9992). The TPC (mg·g^−1^) was determined using this standard curve method.

### 2.5. Determination of Total Flavonoid Content (TFC)

The method described by Yan et al. [15] and Li et al. [16] was used to determine the TFC. Sample extracts were prepared according to the methodology outlined in Section 2.3. In detail, 1 mL of the extract was precisely transferred into a 10 mL stoppered colorimetric tube. To this, 2 mL of 0.1 mol·L^−1^ AlCl_3_ solution was introduced, and then 3 mL of a 1 mol·L^−1^ CH_3_COOK solution was introduced. The mixture was subsequently diluted to the 10 mL mark using 70% methanol and mixed thoroughly. Following a reaction period of 30 min, absorbance was recorded at 420 nm with an Ultraviolet-Visible spectrophotometer (A380, Aoyi Instruments Co., Ltd., Shanghai, China).

To construct the standard curve, aliquots (0, 0.5, 1.0, 2.0, 3.0, and 4.0 mL) of a 0.05 mg·mL^−1^ rutin standard solutions were pipetted into a series of 10 mL graduated, stoppered colorimetric tubes. The color development and absorbance measurement were performed following the aforementioned procedure. Subsequently, the standard curve was constructed, giving the regression equation: Y = 2.9673x − 0.0089 (R^2^ = 0.9994). This equation was used to quantify TFC (mg·g^−1^).

### 2.6. Determination of Polysaccharide Content

In centrifuge tubes, 1 g sample was combined with methanol in a 1:50 (w·v^−1^) proportion and treated ultrasonically processing at 40 °C for 30 min. After sonication, the mixture was centrifuged at 10,000 r·min^−1^ at 4 °C for 15 min to isolate the supernatant. This extraction procedure was carried out two additional times identically. The pooled supernatants from all cycles were evaporated at 40 °C and reconstituted with methanol to 25 mL, yielding the final sample extracts, which were placed at 4 °C pending subsequent analysis.

Polysaccharide quantification was performed according to the method described by Birhanie et al. [17]. In summary, 0.5 mL of extract was combined with 0.5 mL of a 10% phenol solution, after which 5 mL of sulfuric acid was gradually introduced. The resulting mixture underwent heating in a 40 °C water bath for 10 min, followed by cooling to ambient temperature. Absorbance readings were taken at 490 nm with an Ultraviolet-Visible spectrophotometer (A380, Aoyi Instruments Co., Ltd., Shanghai, China). A series of D-(+)-anhydroglucose solutions, with concentrations of 20 to 200 μg·mL^−1^, were analyzed identically to generate the calibration curve. The regression equation derived was Y = 0.0063x + 0.0295 (R^2^ = 0.9985), enabling the calculation of polysaccharide content (mg·g^−1^).

### 2.7. DPPH Scavenging Capacity Assay

In anhydrous ethanol, a DPPH solution with a concentration of 2 × 10^−4^ mol·L^−1^ was formulated. Sample extracts were obtained in accordance with the protocol in Section 2.3. Absorbance values were recorded on an Ultraviolet-Visible spectrophotometer (A380, Aoyi Instruments Co., Ltd., Shanghai, China), and the DPPH radical scavenging rate was derived by means of the given Equation (1) [18]:

(1) $$K=\frac{D_{1}-D_{2}}{D_{3}}\;\times \;100$$

In this equation, D_1_ represents the absorbance at 515 nm of a blend containing 2 mL DPPH solution and 2 mL sample extract at concentrations ranging from 0.2 to 5 μg·mL^−1^; D_2_ denotes the absorbance of a mixture with 2 mL sample extract (at corresponding concentrations) and 2 mL of 70% methanol; the absorbance of a 2 mL DPPH solution combined with 2 mL 70% methanol is denoted as D_3_. The IC_50_ value (mg·mL^−1^)—the sample concentration necessary to reach 50% scavenging activity—was obtained by fitting the scavenging rate to sample concentration. The precision of IC_50_ values was confirmed by the high coefficient of determination (R^2^ > 0.95) of all fitted curves. The same with ABTS radical scavenging capacity assay and the determination of α-glucosidase inhibition capacity.

### 2.8. ABTS Radical Scavenging Capacity Assay

The ABTS radical scavenging activity was assessed with minor modifications to the procedure of Tao et al. [19]. Specifically, 7 mmol·L^−1^ ABTS solution was combined in a 1:1 volumetric ratio with 2.45 mmol·L^−1^ potassium persulfate in an amber vessel to prepare the working solution, which was then left to react in darkness for 12 h. Before the assay, the ABTS working solution was adjusted with distilled water to an absorbance value of 0.70 ± 0.20 measured at 734 nm. Sample extracts were generated as detailed in Section 2.3.

Absorbance measurements were conducted using an Ultraviolet-Visible spectrophotometer (A380, Aoyi Instruments Co., Ltd., Shanghai, China). The ABTS radical scavenging capacity was computed according to Equation (2):

(2) $$C=\frac{1-\left(B_{1}-B_{2}\right)}{B_{3}}\;\times \;100$$

Here, B_1_ represents the absorbance at 734 nm of a blend containing 2 mL distilled water and 4 mL ABTS working solution, measured after reaction for 10 min; B_2_ corresponds to the absorbance of a mixture comprising 2 mL sample extract and 4 mL ABTS working solution following the same reaction period; B_3_ refers to the absorbance of a combination of 2 mL sample extract and 4 mL distilled water after 10 min. By plotting the ABTS scavenging rate against the concentration of each sample, the concentration required for each sample to achieve a 50% ABTS scavenging rate (i.e., the IC_50_ value (mg·mL^−1^)) can be determined.

### 2.9. Determination of α-Glucosidase Inhibition Capacity

The α-glucosidase inhibitory assay was conducted based on a previously reported approach by Xie et al. [20] with slight modifications. Following the sample preparation protocol in Section 2.3, the experimental procedure was carried out as follows: into centrifuge tubes were added 0.1 mL of sample extract, 0.1 mL of 0.1 mol·L^−1^ phosphate buffer (pH 6.9), and 0.1 mL of 0.5 U·mL^−1^ α-glucosidase solution in sequence. Subsequently, 0.2 mL of 3 mmol·L^−1^ PNPG substrate was introduced. Following a 20 min incubation at 37 °C in a water bath, the mixture had its reaction halted with 5 mL of 1 mol·L^−1^ Na_2_CO_3_ solution. Absorbance was recorded at 405 nm using an Ultraviolet-Visible spectrophotometer (A380, Aoyi Instruments Co., Ltd., Shanghai, China). The inhibition percentage was calculated according to Equation (3):

(3) $$I\left(\%\right)=\left[1-\frac{{(A}_{3}-A_{2})}{{(A}_{1}-A_{0})}\right]\times 100$$

In this equation, the blank control is denoted as A_0_, A_1_ represents the control reaction, A_2_ indicates the sample blank, and A_3_ signifies the sample reaction group. By plotting the α-glucosidase Inhibition rate against the concentration of each sample, the concentration required for each sample to achieve a 50% α-glucosidase Inhibition rate (i.e., the IC_50_ value (mg·mL^−1^)) can be determined.

### 2.10. Metabolomics Analysis

Metabolites were extracted from the samples following the method described by Dunn et al. [21]. Metabolomic profiling was conducted on 3 biological replicates per group. Each replicate represents an independent fungal culture. For each specimen, an aliquot of 50 mg was precisely weighed and transferred into 1000 μL of extraction solution spiked with 20 mg·L^−1^ internal standard, then vortexed for 30 s. After adding steel balls, the mixture was processed in a 45 Hz grinder for 10 min, followed by 10 min of ultrasonication in an ice-water bath.

Chromatographic separation occurred on a Waters Acquity UPLC HSS T3 column (1.8 μm, 2.1 × 100 mm), which was kept at 40 °C, with a mobile phase flow rate of 0.3 mL·min^−1^ and an injection volume of 2 μL [22]. Detection via mass spectrometry was performed using electrospray ionization (ESI) in both positive and negative ion modes [22]. Capillary voltage settings were 2500 V for positive and −2000 V for negative mode. The ion source temperature was held at 100 °C, and the acquisition mass range covered m·z^−1^ 50–1200.

Raw data acquired with MassLynx V4.2 were processed through Progenesis QI V4.0 software to perform peak picking, alignment, and related procedures. Metabolite identification was further conducted by querying the online METLIN database, public databases, and Biomarker Technologies (BMK) self-built database via Progenesis QI, accompanied by theoretical fragment ion verification.

To assess the consistency of biological replicates, Spearman rank correlation coefficients were calculated for all pairwise comparisons between samples. A correlation coefficient (r) closer to 1 indicates a stronger similarity between replicates. High correlation within experimental groups relative to between groups ensures the reliability of subsequent differential analysis.

### 2.11. Statistical Analysis

All quantitative data are expressed as mean ± standard deviation (on a dry weight basis) of triplicate measurements. Data were analyzed using one-way analysis of variance (ANOVA), and the means were compared using Tukey’s honestly significant difference post hoc test at a significance level of *p* < 0.05 using IBM SPSS Statistics 26.0 (SPSS Inc., Chicago, IL, USA). Data visualization was performed using Origin 2021 (OriginLab, Northampton, MA, USA). Metabolomics analyses were performed using the BMK Cloud Platform (www.biocloud.net (accessed on 31 July 2025)). Specifically: Principal Component Analysis (PCA) was conducted on the variance-stabilized transformed data to visualize metabolite segregation. Clustering heat maps were generated using the complete linkage method with Euclidean distance based on the Z-score normalized abundance of significantly altered metabolites. Volcano plots were created to visualize differential metabolites, with significance thresholds set at *p* < 0.05 and fold change > 1.2. KEGG enrichment analysis was carried out on the significant differential metabolites using a hypergeometric test, with pathways considered significantly enriched at a Benjamini–Hochberg corrected *p*-value < 0.1.

## 3. Results and Discussions

This section presents the comprehensive effects of SSF with three edible-medicinal fungi (*A. auricula*, *G. lucidum*, and *H. erinaceus*) on the functional constituents and bioactivities of Tartary buckwheat. The analysis begins with changes in fundamental bioactive compounds (total phenols, total flavonoids and polysaccharide), proceeds to the consequent impacts on in vitro antioxidant and α-glucosidase inhibitory activities, and concludes with alterations in specific metabolites revealed by non-targeted metabolomics.

### 3.1. The Influence of SSF on the Content of Bioactive Substances in Tartary Buckwheat

Phenolic compounds, flavonoids, and polysaccharides are key bioactive substances in Tartary buckwheat, primarily contributing to its antioxidant and other functional activities. In this study, the contents of these components in NFTB, AFTB, GFTB, and HFTB were determined, with the results presented in Table 1. The impact of SSF on the bioactive substances of Tartary buckwheat varied depending on the fungal strain and the specific compound. Compared with NFTB, the total phenol content of AFTB, GFTB, and HFTB significantly increased (*p* < 0.05). However, for total flavonoid content, a significant increase was only observed in GFTB, whereas AFTB led to a significant decrease. Regarding polysaccharide content, both AFTB and GFTB showed a significant increase, but no significant enhancement was observed in HFTB. Total flavonoids in GFTB increased by 13.65%, while polysaccharide contents in AFTB and GFTB rose by 53.98% and 26.08%, respectively. The above results are consistent with those found by Zhang et al. [23].

### 3.2. Alterations in In Vitro Antioxidant Activity

**Figure 1:**
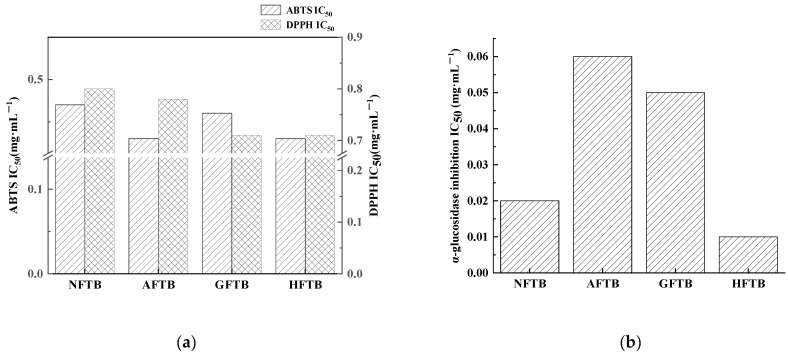
Changes in functional activities. (**a**) In vitro antioxidant activity (DPPH and ABTS Radical Scavenging Activities by IC_50_); (**b**) α-glucosidase inhibitory activity by IC_50_. IC_50_ values are presented as point estimates derived from non-linear regression of dose–response curves based on triplicate measurements (*n* = 3). Non-fermented Tartary buckwheat (NFTB), *A. auricula*-fermented Tartary buckwheat (AFTB), *G. lucidum*-fermented Tartary buckwheat (GFTB), and *H. erinaceus*-fermented Tartary buckwheat (HFTB).

The assessment of in vitro antioxidant properties was conducted through scavenging assays targeting DPPH and ABTS radicals. As shown in Figure 1a, following SSF with three fungi, the IC_50_ values related to DPPH and ABTS radical scavenging activities of Tartary buckwheat were lowered. The greatest decrease was found in HFTB, where the IC_50_ values dropped by 0.09 mg·mL^−1^ and 0.04 mg·mL^−1^ for DPPH and ABTS assays, respectively. These changes reflect an enhancement in scavenging ability of 11.25% and 8.51%. The findings indicate that SSF using edible-medicinal fungi improves the antioxidant properties of Tartary buckwheat, aligning with the results reported by Li et al. [24]. In addition, a more potent antioxidant effect was exhibited by HFTB compared to GFTB and AFTB.

### 3.3. Alterations in α-Glucosidase Inhibitory Activity

Tartary buckwheat contains various bioactive substances that are credited with hypoglycemic activity [25]. Under the experimental conditions, the α-glucosidase inhibitory activity of AFTB and GFTB declined, whereas the HFTB sample demonstrated a 50% increase, as illustrated in Figure 1b. These outcomes are in agreement with the results from Zhou et al. [26], indicating that *H. erinaceus* is a more appropriate fungal strain for improving the α-glucosidase inhibitory potential of Tartary buckwheat.

A methodological limitation of this study must be considered regarding the α-glucosidase inhibition assay. The assay was conducted using the crude 70% methanol extract. It is well established that methanol can denature enzymes and inhibit their activity. Therefore, the inhibitory activity observed here may be partially attributable to the solvent. Although the dose-dependent response and the known inhibitory compounds identified in our extract by UPLC-MS suggest genuine bioactive potential, and additional experiments indicate that using 70% methanol as the extraction solvent has only a minor impact on the results, the exact contribution of the metabolites themselves remains to be quantified. Future work will necessitate the evaporation of methanol and re-constitution of the extract in a compatible solvent system, such as aqueous buffer with minimal DMSO, to conclusively validate the intrinsic α-glucosidase inhibitory activity of solid-state fermented Tartary buckwheat.

### 3.4. Metabolomics Analysis

#### 3.4.1. PCA

Metabolite variations in Tartary buckwheat before and after fermentation were investigated through an untargeted LC-MS metabolomics approach, supported by multivariate statistical analysis. Tentative identification and structural characterization of the compounds were carried out using high-resolution UPLC-ESI-MS under both positive and negative ionization modes. The results revealed that SSF with *A. auricula*, *H. erinaceus*, and *G. lucidum* induced significant changes in the metabolomic profiles of Tartary buckwheat. Additionally, distinct differences were observed among the three fungal strains.

**Figure 2:**
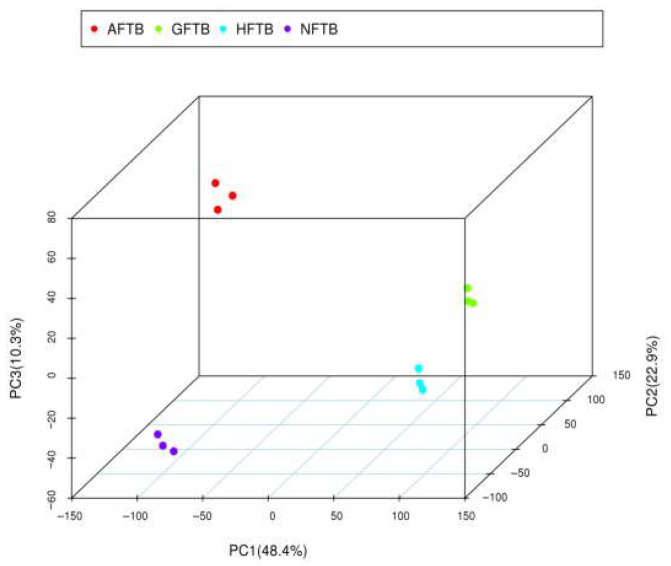
PCA plot of non-fermented Tartary buckwheat (NFTB), *A. auricula*-fermented Tartary buckwheat (AFTB), *G. lucidum*-fermented Tartary buckwheat (GFTB), and *H. erinaceus*-fermented Tartary buckwheat (HFTB) samples.

The PCA plot illustrating the relationships among NFTB, AFTB, GFTB, and HFTB is presented in Figure 2. The combined contribution of PC1, PC2, and PC3 to the total variance was 81.6%, as determined by their respective variances. In the PCA score plot, data points corresponding to each sample group formed tight clusters, demonstrating excellent reproducibility. Furthermore, a clear separation was observed among the various sample groups, as evidenced by substantial inter-group distances. This pattern indicates that SSF of Tartary buckwheat using edible-medicinal fungi substantially modified its metabolite profile, with the extent of modification varying according to the fungal species employed.

#### 3.4.2. Analysis of Differential Metabolites Before and After SSF

The initial annotation of differential metabolites was carried out by matching retention time, precise mass, molecular formula, and MS/MS fragmentation patterns against available databases and established literature. (e.g., KEGG, Lipidmaps, and HMDB). Differential metabolites among samples were screened using the criteria of *p* < 0.05, Variable Importance in the Projection (VIP) > 1, and fold change (FC) > 1.2. Specifically, compared with NFTB, AFTB, GFTB, and HFTB exhibited 694, 1969, and 2130 differential metabolites, respectively. This indicates that SSF with *G. lucidum* and *H. erinaceus* exerts a more profound impact on the metabolism of Tartary buckwheat than that with *A. auricula*.

**Figure 3:**
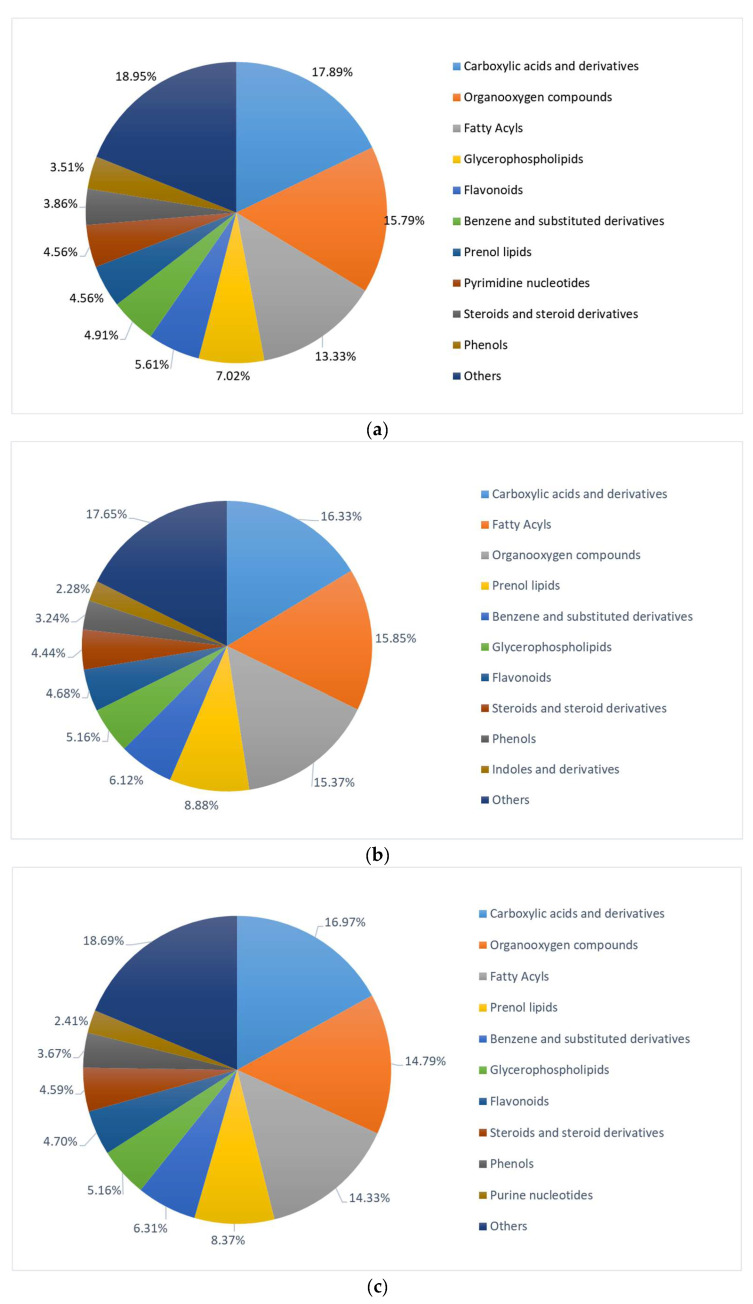
Classification of differential metabolites of AFTB vs. NFTB (**a**), GFTB vs. NFTB (**b**) and HFTB vs. NFTB (**c**). Non-fermented Tartary buckwheat (NFTB), *A. auricula*-fermented Tartary buckwheat (AFTB), *G. lucidum*-fermented Tartary buckwheat (GFTB), and *H. erinaceus*-fermented Tartary buckwheat (HFTB).

All differential metabolites were categorized into 11 major classes (Figure 3), revealing significant differences in the types of differential metabolites produced in Tartary buckwheat via SSF with different edible-medicinal fungi. For AFTB, the most diverse metabolites included carboxylic acids and derivatives (17.89%), organooxygen compounds (15.79%), fatty acyls (13.33%), glycerophospholipids (7.02%), and flavonoids (5.61%). In contrast, the top nine classes of differential metabolites in GFTB and HFTB showed essentially identical types and proportions, suggesting that the metabolic profiles of Tartary buckwheat fermented by *G. lucidum* and *H. erinaceus* are relatively similar but significantly distinct from those fermented by *A. auricula*.

**Figure 4:**
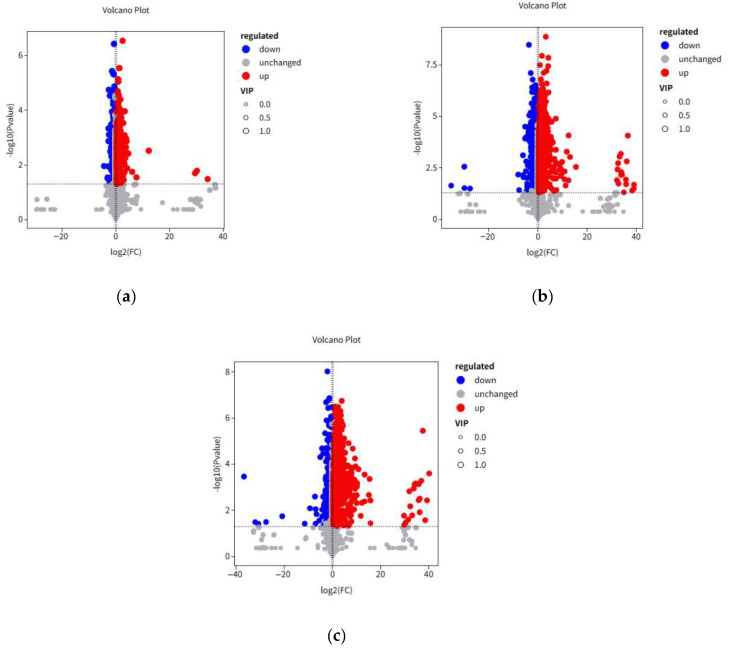
Volcano plot of the differential metabolites of AFTB vs. NFTB (**a**), GFTB vs. NFTB (**b**), and HFTB vs. NFTB (**c**). Each dot represents a metabolite. Non-fermented Tartary buckwheat (NFTB), *A. auricula*-fermented Tartary buckwheat (AFTB), *G. lucidum*-fermented Tartary buckwheat (GFTB), and *H. erinaceus*-fermented Tartary buckwheat (HFTB).

According to the volcano plots, SSF with *A. auricula* led to the significant up-regulation of 302 metabolites and down-regulation of 392. In contrast, the GFTB group (Figure 4b) exhibited a more pronounced response, with 868 and 1101 metabolites being significantly up- and down-regulated, respectively. Metabolite profiling of HFTB revealed 766 substances with significant up-regulation and 1364 with significant down-regulation (Figure 4c).

#### 3.4.3. Flavonoids and Phenols Compounds Analysis

**Figure 5:**
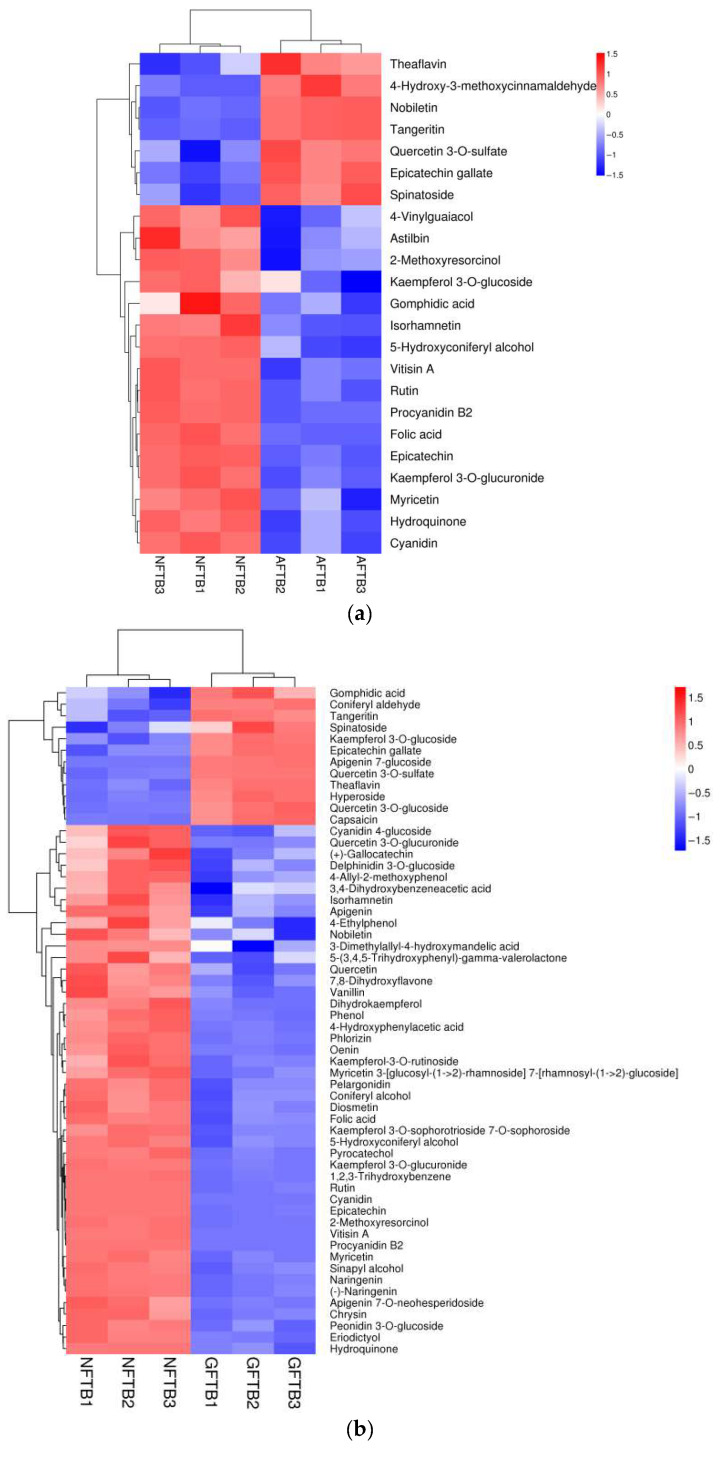

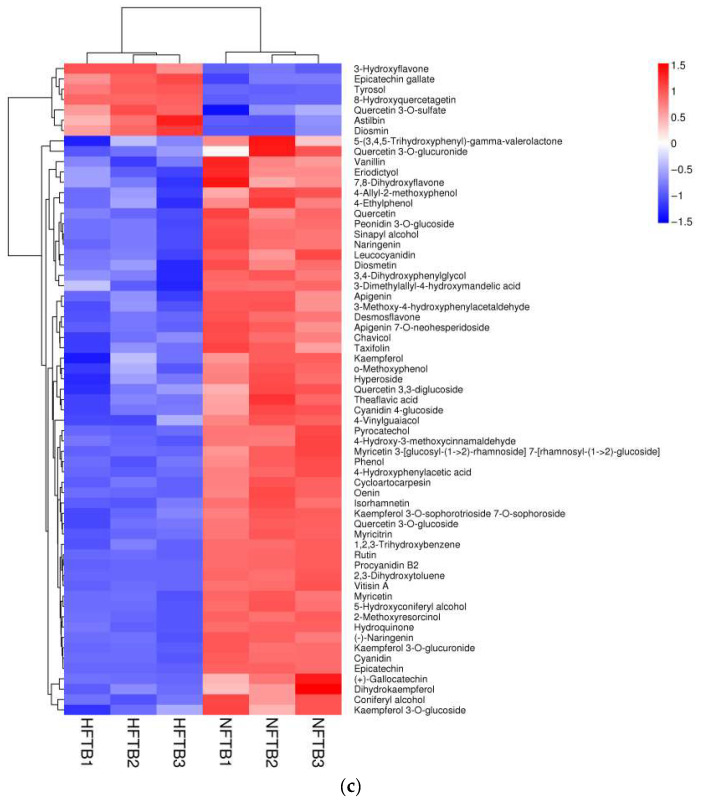
Heat map of phenols and flavonoids of AFTB vs. NFTB (**a**), GFTB vs. NFTB (**b**), and HFTB vs. NFTB (**c**). Non-fermented Tartary buckwheat (NFTB), *A. auricula*-fermented Tartary buckwheat (AFTB), *G. lucidum*-fermented Tartary buckwheat (GFTB), and *H. erinaceus*-fermented Tartary buckwheat (HFTB).

Phenolic and flavonoids compounds represent the key bioactive constituents in Tartary buckwheat. As depicted in Figure 5, a total of 26 phenolic and flavonoid compounds were detected in AFTB, among which 7 were significantly up-regulated. Specifically, nobiletin, tangeritin, 4-Hydroxy-3-methoxycinnamaldehyde, epicatechin gallate, quercetin 3′-O-sulfate, spinatoside, and theaflavin exhibited marked up-regulation. However, most phenolic substances and half of the flavonoids, including kaempferol 3-O-glucoside, rutin, kaempferol 3-O-glucuronide, and astilbin were significantly down-regulated. Similarly, 58 phenolic and flavonoid were detected in the GFTB, with 12 showing significant up-regulation. These included three phenolic compounds (coniferyl aldehyde, gomphidic acid and capsaicin) and nine flavonoids (apigenin 7-glucoside, quercetin 3′-O-sulfate, theaflavin and kaempferol 3-O-glucoside, hyperoside, epicatechin gallate, quercetin 3-O-glucoside, tangeritin and spinatoside). Finally, 63 phenolic and flavonoid compounds were detected in HFTB, of which 7 were significantly up-regulated.

#### 3.4.4. Analysis of Flavonoids

Flavonoids are recognized as crucial bioactive components in Tartary buckwheat. A total of 59 differential flavonoid metabolites were annotated and classified in three SSF samples, mainly categorized into flavans, flavones, hydroxyflavonoids, O-methylated flavonoids, bioflavonoids, and polyflavonoids, flavonoid glycosides, pyranoflavonoids and isoflavones. Detailed results are presented in Table 2.

The flavonoid profile of Tartary buckwheat is characterized by a high abundance of rutin, quercetin, nicotiflorin, and kaempferol. These four compounds alone constitute 73.05–81.79% of the total flavonoids. Other metabolites present in notable concentrations include quercimeritrin, hesperidin, procyanidin B2, epicatechin, narcissin, catechin, and astragalin [27]. In our study, rutin, quercetin, kaempferol, procyanidin B2, and epicatechin all exhibited a down-regulated trend, which might be attributed to the transformation induced by the SSF process. Among the up-regulated flavonoids, flavonoid glycosides, flavones, and flavans constituted the majority.

Through a comparative study of SSF employing three edible-medicinal fungi, it was found that metabolites such as epicatechin gallate, theaflavin, quercetin 3′-O-sulfate, tangeritin, and spinatoside were significantly up-regulated in two or three of the fermented samples. The majority of the remaining metabolites were significantly down-regulated, which might be attributed to their conversion into other substances during the SSF process [6].

Epicatechin gallate (ECG) was significantly up-regulated in all three samples. This finding is of particular significance because ECG is well-documented in the literature for its potent antioxidant capacity [28,29], attributed to its molecular structure rich in phenolic hydroxyl groups that readily donate hydrogen atoms to stabilize free radicals [30]. For instance, existing literature reports that the ECG content in black tea correlates strongly with its potentiated antioxidant activity [31]. Therefore, the remarkable up-regulation in ECG identified in our metabolomic data provides a compelling biochemical explanation for the concurrent enhancement in free-radical scavenging activities observed in the fermented samples. Our discovery highlights the potential of SSF as a bioprocessing strategy to enhance the relative levels of specific bioactive compounds, such as ECG, in our substrate.

In GFTB, quercetin 3′-O-sulfate was increased by 10.01-fold, far exceeding that in AFTB (1.58-fold) and HFTB (1.42-fold). Quercetin 3′-O-sulfate, a sulfated derivative formed by the addition of a sulfate group to the 3′-hydroxyl position on quercetin’s B ring, was found to be synthesized more effectively by *G. lucidum* than by *A. auricula* and *H. erinaceus*, as indicated by these results. It exhibits multiple health-promoting properties, including a stronger free radical scavenging activity than well-recognized antioxidants such as alpha-tocopherol [32], as well as an ability to counteract peroxynitrite-induced oxidative hepatotoxicity effectively [33]. In metabolic studies, quercetin sulfate derivatives are utilized as key reference compounds, while sulfated metabolites are recognized for their strong antioxidant properties [34]. Notably, quercetin-3′-O-sulfate—a water-soluble form derived from quercetin, the predominant and highly active flavonoid antioxidant—is of particular interest [35].

The distinct flavonoid profile observed in GFTB can be attributed to comprehensive transformations mediated by the microbial consortium during solid-state fermentation (SSF). The initial and critical step likely involves the microbially driven hydrolysis of rutin. This de-glycosylation reaction, catalyzed by microbial β-glucosidases, cleaves the rutinoside moiety to yield the aglycone quercetin, explaining the concurrent down-regulation of rutin and the up-regulation of quercetin metabolites—a finding consistent with Lukšič et al. [36]. Subsequently, the liberated quercetin undergoes extensive microbial glycosylation. This re-glycosylation, facilitated by glycosyltransferases, conjugates various sugar donors (e.g., UDP-glucose) to specific hydroxyl groups on the quercetin backbone, generating a spectrum of water-soluble glycosylated metabolites such as quercetin 3-O-glucoside, quercetin 3,3′-diglucoside, and quercetin 3-O-glucuronide. The significant up-regulation of quercetin 3-O-glucoside (FC = 1.49) in GFTB (Table 2) provides direct evidence for this active process. An analogous biosynthetic pathway is evident for kaempferol, with marked up-regulation of both kaempferol 3-O-glucoside (FC = 2.82) and kaempferol 3-O-rutinoside (FC = 1.49). These results collectively demonstrate that microbial glycosylation of flavonoids was markedly more active in GFTB than in AFTB and HFTB, fundamentally enhancing their water solubility and potential bioavailability.

Isoflavones are generally considered to exist only in certain leguminous plants; however, in our research, nine types of isoflavones were detected, which is consistent with the results reported by Ke et al. [27] and Li et al. [37]. In GFTB, kievitone hydrate (FC = 1.74) was significantly up-regulated, whereas daidzin, daidzein, rotenone, medicarpin, (-)-medicarpin, and genistein were significantly down-regulated. In HFTB, pseudobaptigenin (FC = 1105.35) was significantly up-regulated, while daidzin, daidzein, medicarpin, (-)-medicarpin, 6″-O-acetylglycitin, and genistein were significantly down-regulated. No isoflavones were detected in AFTB.

Differential metabolites unique to HFTB, such as 3-hydroxyflavone, 8-hydroxyquercetagetin, diosmin, and astilbin, have been demonstrated to possess significant antioxidant activity [38,39,40,41]. Thus, the superior antioxidant performance of HFTB can be attributed, at least in part, to the aforementioned findings.

#### 3.4.5. KEGG Metabolic Pathway Analysis

**Figure 6:**
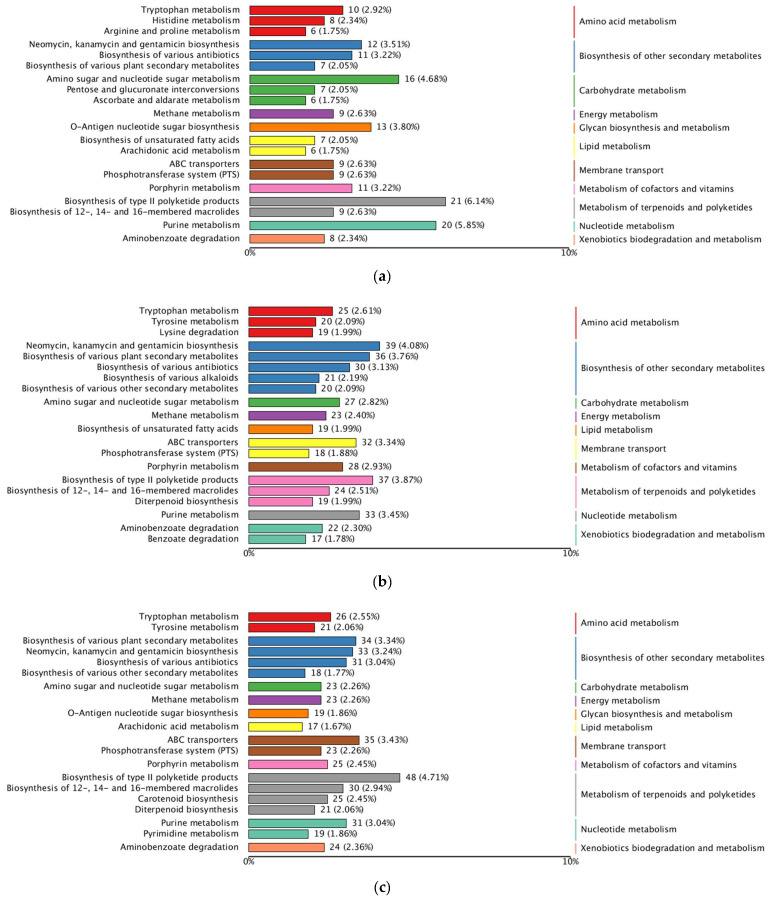
KEGG enrichment analysis of differential metabolites of AFTB vs. NFTB (**a**), GFTB vs. NFTB (**b**), and HFTB vs. NFTB (**c**). Non-fermented Tartary buckwheat (NFTB), *A. auricula*-fermented Tartary buckwheat (AFTB), *G. lucidum*-fermented Tartary buckwheat (GFTB), and *H. erinaceus*-fermented Tartary buckwheat (HFTB).

To gain deeper insights into the metabolic pathways linked to differential metabolites in Tartary buckwheat during solid-state fermentation with *A. auricula*, *H. erinaceus*, and *G. lucidum*, a KEGG pathway enrichment analysis was conducted on the identified metabolites from every sample. The results are presented by Figure 6. As observed, the key metabolic pathways vary among Tartary buckwheat samples fermented by different fungi.

Among all the samples, the pathways with the largest number of enriched differential metabolites are biosynthesis of other secondary metabolites and metabolism of terpenoids and polyketides. The significant enrichment of the “biosynthesis of other secondary metabolites” pathway across all three fermented samples indicates that SSF strongly activates diverse secondary metabolic processes. While this pathway includes the enrichment of certain flavonoids, its breadth signifies a fundamental shift in the metabolic profile. Specifically, SSF not only modifies the inherent flavonoids of Tartary buckwheat but also leads to the synthesis of a unique set of fungal-derived secondary metabolites. This expansion of chemical diversity directly enhances the functional value of the fermented product of Tartary buckwheat.

Additionally, in amino acid metabolism, tryptophan metabolism was detected in all three samples. Additionally, histidine metabolism, as well as arginine and proline metabolism, were identified in AFTB. GFTB exhibited tyrosine metabolism and lysine metabolism, while HFTB showed tyrosine metabolism. Tryptophan and lysine are essential amino acids for humans, and their presence enhances the nutritional value of Tartary buckwheat. Amino sugar and nucleotide sugar metabolism were present in all three samples. However, AFTB displayed a more diverse range of carbohydrate metabolism pathways, which also included pentose and glucuronate interconversions, as well as ascorbate and aldarate metabolism.

## 4. Conclusions

This study aimed to evaluate the effectiveness of SSF with three edible-medicinal fungi in enhancing the functional value of Tartary buckwheat. Our findings successfully demonstrates that SSF is an effective strategy. All three fungi enhanced the antioxidant activity of Tartary buckwheat, a 50% enhancement in α-glucosidase inhibitory activity was observed under the tested conditions, which can be attributed to SSF employing *H. erinaceus*. Non-targeted metabolomics profiling demonstrated that the metabolite profile of Tartary buckwheat was substantially modified through SSF with various edible-medicinal fungi, with the degree of alteration being strain-dependent. The significant up-regulation of ECG in all samples and the distinctly higher quercetin 3′-O-sulfate level in GFTB contribute to the altered functional activities of Tartary buckwheat after SSF. The biosynthesis of other secondary metabolites and terpenoid and polyketide metabolism emerged as the two most active pathways.

In summary, this work confirms that SSF can successfully augment the bioactive components and health-related functions of Tartary buckwheat. A limitation of this study is that only in vitro activity was assessed. Future research should include in vivo experiments to validate the physiological efficacy.

## Figures and Tables

**Table 1:** Contents of bioactive substances in samples.

Bioactive Substances	NFTB	AFTB	GFTB	HFTB
Total phenol (mg·g^−1^)	10.02 ± 0.03 ^c^	10.54 ± 0.11 ^b^	10.73 ± 0.05 ^a^	10.71 ± 0.03 ^ab^
Total flavonoid (mg·g^−1^)	14.21 ± 0.12 ^b^	13.30 ± 0.08 ^c^	16.15 ± 0.12 ^a^	14.43 ± 0.20 ^b^
Polysaccharide (mg·g^−1^)	131.43 ± 0.34 ^c^	202.38 ± 1.01 ^a^	165.71 ± 0.34 ^b^	128.33 ± 0.67 ^d^

Values were expressed as mean ± standard deviation of three determinations. Values followed by different superscript small letters (a–d) are remarkably different (*p* < 0.05) among the samples. Non-fermented Tartary buckwheat (NFTB), *A. auricula*-fermented Tartary buckwheat (AFTB), *G. lucidum*-fermented Tartary buckwheat (GFTB), and *H. erinaceus*-fermented Tartary buckwheat (HFTB).

**Table 2:** The fold change values of differential metabolites of flavonoids.

Name	AFTB	GFTB	HFTB
**Flavans**			
Eriodictyol	-	0.47	0.81
(-)-Naringenin	-	0.19	0.54
Naringenin	-	0.21	0.58
Leucocyanidin	-	-	0.80
Theaflavic acid	-	-	0.72
Epicatechin	0.51	0.06	0.29
(+)-Gallocatechin	-	0.70	0.65
Dihydrokaempferol	-	0.25	0.60
Taxifolin	-	-	0.13
Epicatechin gallate	1.75	1.52	1.45
Theaflavin	1.42	4.46	-
**Flavones**			
Kaempferol	-	-	0.83
Myricetin	0.81	0.68	0.67
3-Hydroxyflavone	-	-	1.21
Quercetin	-	0.76	0.68
Isorhamnetin	0.79	0.83	0.56
7,8-Dihydroxyflavone	-	0.67	0.79
Quercetin 3′-O-sulfate	1.58	10.01	1.42
8-Hydroxyquercetagetin	-	-	3.20
Apigenin	-	0.78	0.72
Chrysin	-	0.55	-
**Hydroxyflavonoids**			
Cyanidin	0.74	0.14	0.44
Pelargonidin	-	0.83	-
**O-methylated flavonoids**			
Desmosflavone	-	-	0.80
Diosmetin	-	0.78	0.77
Nobiletin	11.45	0.60	-
Tangeritin	11.09	1.34	-
**Biflavonoids and polyflavonoids**			
Procyanidin B2	0.51	0.13	0.32
Vitisin A	0.49	0.18	0.33
**Flavonoid glycosides**			
Diosmin	-	-	1.23
Quercetin 3-O-glucoside	-	1.49	0.82
Oenin	-	0.81	0.78
Kaempferol 3-O-sophorotrioside 7-O-sophoroside	-	0.80	0.78
Myricetin 3-[glucosyl-(1->2)-rhamnoside] 7-[rhamnosyl-(1->2)-glucoside]	-	0.72	0.70
Quercetin 3,3′-diglucoside	-	-	0.77
Cyanidin 4′-glucoside	-	0.78	0.67
Quercetin 3-O-glucuronide	-	0.47	0.50
Kaempferol 3-O-glucoside	0.51	2.82	0.39
Hyperoside	-	1.83	0.78
Myricitrin	-	-	0.75
Peonidin 3-O-glucoside	-	0.81	0.79
Apigenin 7-O-neohesperidoside	-	0.81	0.77
Rutin	0.74	0.22	0.56
Kaempferol 3-O-glucuronide	0.50	0.20	0.25
Astilbin	0.82	-	1.39
Phlorizin	-	0.82	-
Kaempferol-3-O-rutinoside	-	1.49	-
Spinatoside	1.50	1.22	-
Delphinidin 3-O-glucoside	-	0.79	-
Apigenin 7-glucoside	-	3.57E + 11	-
**Pyranoflavonoids**			
Cycloartocarpesin	-	-	0.82
**Isoflavones**			
Medicarpin	-	0.82	0.83
(-)-Medicarpin	-	0.64	0.71
Daidzin	-	0.72	0.74
Daidzein	-	0.34	0.64
Genistein	-	0.50	0.55
Kievitone hydrate	-	1.74	-
6″-O-Acetylglycitin	-	-	0.66
Pseudobaptigenin	-	-	1105.35

“-“: No such differential metabolite in the sample. Fold change values are unitless ratios calculated as the mean normalized intensity in the treatment group divided by the mean normalized intensity in the control group. Non-fermented Tartary buckwheat (NFTB), *A. auricula*-fermented Tartary buckwheat (AFTB), *G. lucidum*-fermented Tartary buckwheat (GFTB), and *H. erinaceus*-fermented Tartary buckwheat (HFTB).

## Data Availability

The original contributions presented in the study are included in the article, further inquiries can be directed to the corresponding author.

## Abbreviations

The following abbreviations are used in this manuscript:

SSF	Solid-state fermentation
PDA	Potato dextrose agar
PNPG	p-Nitrophenyl-β-d-Galactopyranoside
ABTS	3-ethylbenzthiazoline-6-sulphonic acid
DPPH	1,1-Diphenyl-2-picrylhydrazyl
AFTB	*A. auricula*-fermented Tartary buckwheat
GFTB	*G. lucidum*-fermented Tartary buckwheat
HFTB	*H. erinaceus*-fermented Tartary buckwheat
NFTB	Non-fermented Tartary buckwheat
PCA	Principal component analysis
KEGG	Kyoto Encyclopedia of Genes and Genomes
FC	Fold change
VIP	Variable Importance in the Projection
ECG	Epicatechin gallate
CICC	Center of Industrial Culture Collection
LC-MS	Liquid Chromatography–Mass Spectrometry
UPLC	Ultra-performance liquid chromatography
